# Think Outside the Box: A Unique Approach to the Management of Comorbid Malignant Pleural and Pericardial Effusions

**DOI:** 10.1016/j.chpulm.2025.100179

**Published:** 2025-05-17

**Authors:** Mark Gartner, Steven Stoffel, Nicholas Villalobos, Whittney Warren

**Affiliations:** Department of Medicine, Division of Pulmonology and Critical Care Medicine, Brooke Army Medical Center, JBSA Ft Sam Houston, TX

A 42-year-old man with recently diagnosed stage IV lung adenocarcinoma presented to the hospital with dyspnea and presyncope. During his index hospitalization earlier that year, he presented with pericardial tamponade. He was found to have extensive pleural/pericardial metastasis and had previously undergone both a left thoracoscopic pericardial window and placement of a left basilar indwelling pleural catheter (IPC) for outpatient effusion management.

In the emergency department during this visit, he was found to be hypotensive with pulsus paradoxus and echocardiographic findings suggestive of a recalcitrant episode of tamponade (Video 1). His IPC was additionally found to be dysfunctional due to pleurodesis of the surrounding pleura, and CT scan demonstrated interval development of a multiloculated pleural effusion and moderate pericardial effusion (e-Fig 1).

*Questions: What is the most appropriate imaging modality to assess the size/location of his pericardial window and its relationship to his pleural effusion? What management strategies could be implemented in the care of this patient? Can serial thoracentesis safely manage both pleural and postwindow pericardial effusions?*

*Next* *steps:* Although CT scan can assist with this, point-of-care ultrasound (POCUS) is the ideal imaging modality because septations are more readily identified with ultrasound. Note the representative anatomy and direct connection of the pleura and pericardium in Video 2. We can manage the patient with the following strategies: pericardiocentesis, thoracentesis of a connected locule, and repeat thoracic surgery. After evaluation of the CT scan/POCUS, his pericardial window was found to have still an unobstructed connection to a large anterior locule (e-Fig 2, Video 2). The patient underwent thoracentesis on the anterior chest in the third rib space at the midclavicular line leading to the resolution of his anterior pleural/pericardial effusion (Video 3). Since his admission, both his effusions have been managed as an outpatient with serial anterior thoracenteses.

## Discussion

Malignant pleural effusions (MPEs) and malignant pericardial effusions (MPeEs) are common complications in patients with advanced malignancies, particularly lung/breast adenocarcinomas and lymphomas. Development of these effusions are usually related to local invasion of tumor cells within the pleural/pericardial serous membranes and associated inflammation and endothelial/capillary dysfunction. Specifically in lung cancer, MPE is present at the time of diagnosis in approximately 8% to 15% of cases and is expected to develop in 40% to 50% of patients during the disease.^1^ The prevalence of MPeE is less defined in lung cancers but is estimated to be between 8% and 12% in various autopsy studies and is most associated with adenocarcinomas.^2^ Tamponade was the presenting finding in up to 50% of MPeE cases and has high recurrence rates even with therapy.^2^

The presence of these effusions indicates advanced disease and is associated with a poor prognosis. Median survival after the diagnosis of MPE ranges from 3 to 8 months, whereas for MPeE, the median survival is approximately 2 to 6 months, depending on the other case-specific factors and the patient’s overall condition.^3^^,^^4^

The management goals for both effusions are symptom palliation, recurrence risk reduction, and improvement in quality of life. For pericardial effusions causing tamponade, the initial treatment is usually pericardiocentesis, followed by more definitive procedures (eg, creation of a pericardial window to prevent fluid reaccumulation).^5^, ^6^, ^7^ In the case of pleural effusions, thoracentesis can provide temporary relief, but more lasting solutions (eg, placement of an IPC, pleurodesis) are often necessary.^8^^,^^9^

This case highlights multiple anatomic challenges that can be encountered in patients with severe pleural and pericardial metastasis.

The IPC initially placed for the management of pleural and postwindow pericardial effusion became nonfunctional due to the development of loculations in his pleural space and pleurodesis around the catheter. In this case, it led to catheter failure, requiring its removal. Furthermore, despite having a pericardial window, the patient continued to experience recurrent episodes of tamponade. This indicates that the fluid accumulation rate of both his pleura and pericardium was rapid enough to fill his hemithorax and exert enough pressure on his heart to induce tamponade physiology. This complicated ongoing fluid management and necessitated alternative strategies for effusion control. Pleurodesis was considered multiple times during his clinical course but was deferred due to the risk of recurrent tamponade with closure of his pleural space.

**e-Figure 1:**
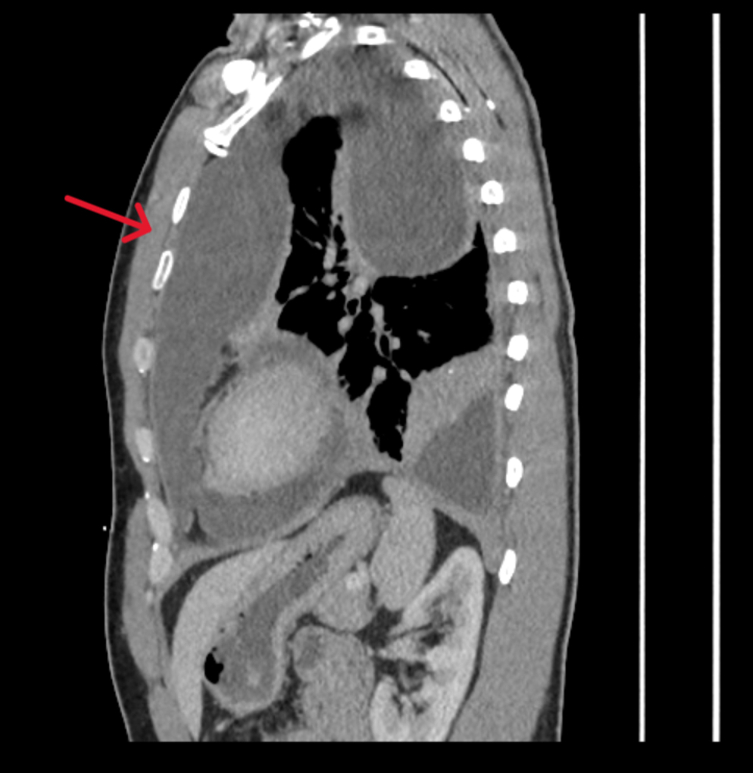
Representative parasagittal cross section taken from the patients CT chest scan demonstrating a multiloculated pleural effusion with regional pleural enhancement and pericardial effusion. The red arrow demarcates the thoracentesis site.

**e-Figure 2:**
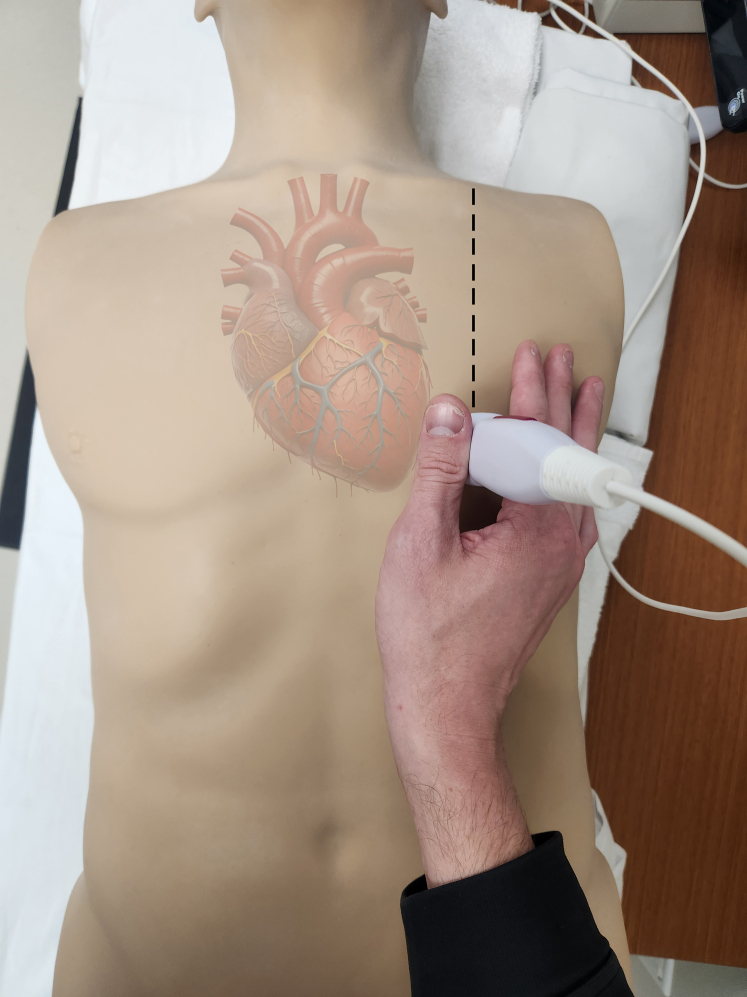
Probe positioning for video 2.

In this case, POCUS was used to identify tamponade, which is a well-described use for this modality. This patient’s point-of-care transthoracic ultrasound windows were additionally able to identify his pericardial window and demonstrate a direct connection between the pericardium and pleural spaces (Video 2) that were free from loculations and allowed for repeated thoracentesis (note this patient’s prethoracentesis and postthoracentesis ultrasounds in Video 3). These can also be identified on his representative CT images (e-Fig 1). Multiple ultrasound views are necessary to fully characterize this relationship and should include the following: parasternal long axis, parasternal short axis, apical 4-chamber, subcostal 4-chamber, and, as in this case, a nonstandard oblique longitudinal parasternal window (e-Fig 2, Video 2).

During his observed clinical course he underwent 15 thoracenteses, with only a single mild pneumothorax that required no additional interventions.

The advanced stage of the underlying malignancy limits the efficacy of treatments aimed at controlling effusions. As the disease progresses, the production of malignant fluid may accelerate, making it increasingly difficult to manage effusions through mechanical drainage alone. Other potential therapeutic options to consider in cases like this are sclerotherapies of the pericardium; although not extensively studied due to the relative paucity of cases, they have been described as safe and effective in case series.^10^

The management of malignant pericardial and pleural effusions in patients with advanced malignancies is complex and requires a multidisciplinary approach. The challenges in this patient’s case included recurrent tamponade despite pericardial window and development of loculations with local pleurodesis around his IPC, highlighting the utility of POCUS as a rapidly deployable modality to identify not only recurrent tamponade, but also safe and adequate drainage sites for repeated thoracenteses, underscoring the importance of individualized care plans.

## Reverberations

1. *Malignant pleural/pericardial effusions are commonly encountered in patients with advanced malignancies and are associated with life-threatening complications, poor quality of life, and poor overall prognosis.*
2. *In patients with both malignant pleural/pericardial effusions who have undergone an ipsilateral pericardial window, serial thoracentesis is a viable treatment option for both effusions.*
3. *Ultrasound can be used successfully to identify the pericardial window and its connection to the pleural space even in complex effusions.*

## Financial/Nonfinancial Disclosures

None declared.

## Supplementary Data

Video 1 A, Parasternal long axis demonstrating moderate to large pericardial effusion. B, Subcostal 4-chamber demonstrating right ventricular diastolic collapse. C, Subcostal IVC long axis demonstrating a plethoric IVC with minimal respirophasic collapse. D, Apical 4-chamber demonstrating both pericardial and loculated pleural effusions. The constellation of these findings is highly suggestive of pericardial tamponade.

Video 2 Longitudinal thoracic ultrasound view taken at the sixth intercostal space along the midclavicular line. This video demonstrates an oblique view of the left ventricular apex featuring the patent pericardial window with an unobstructed pleural/pericardial effusion interface.

Video 3 A, Parasternal long-axis view prethoracentesis. B, Parasternal long-axis view postthoracentesis.
